# Frequency of Gallstones in Patients With Non-alcoholic Fatty Liver Disease

**DOI:** 10.7759/cureus.85508

**Published:** 2025-06-07

**Authors:** Uzma Soomro, Ghulam Abbas, Muhammad Aslam, Sanjay Kirshan Kumar, Ali Hyder, Khaild Tareen, Imran Ahmed, Raja Taha Yaseen Khan, Nasir Hassan Luck

**Affiliations:** 1 General Surgery, Sindh Institute of Medical Sciences, Karachi, PAK; 2 Gastroenterology and Hepatology, Allama Iqbal Medical College, Lahore, PAK; 3 Gastroenterology, Madinah Teaching Hospital, Faisalabad, PAK; 4 Gastroenterology, Ahalia Hospital, Abu Dhabi, ARE; 5 Gastroenterology, Chandka Medical College, Shaheed Mohtarma Benazir Bhutto Medical University, Larkana, PAK; 6 Gastroenterology, Sheikh Khalifa Bin Zayed Al Nahyan Hospital, Quetta, PAK; 7 General Internal Medicine, King’s College Hospital, London, GBR; 8 Hepatogastroenterology, Sindh Institute of Urology and Transplantation, Karachi, PAK

**Keywords:** dyslipidemia, gallstones, metabolic syndrome, non-alcoholic fatty liver disease, pakistan

## Abstract

Introduction

Non-alcoholic fatty liver disease (NAFLD) is a metabolically related liver disease that is becoming increasingly prevalent globally, due to rising obesity, diabetes, and sedentary lifestyles. Concurrently, gallstone disease is a prevalent biliary disease and shares similar risk factors to NAFLD, such as dyslipidemia, insulin resistance, and obesity. In Pakistan, due to a paradigm shift toward urbanization and sedentary lifestyles in recent times, the data are scarce regarding the coexistence of gallstones in the NAFLD population. Therefore, we aimed to find the frequency of gallstones among the NAFLD population and compare the clinical, biochemical, and metabolic profiles of cases of NAFLD presenting with and without gallstones.

Study methodology

A cross-sectional observational study was carried out at the Department of Hepatogastroenterology and General Surgery, Sindh Institute of Urology and Transplantation, Karachi, from January 1, 2023, to June 30, 2023. There were 246 adult patients (age 18 years or above) diagnosed with NAFLD by ultrasound. Those patients having significant alcohol use, pre-existing liver illness, previous cholecystectomy, pregnancy, or drugs that alter the lipid profile were excluded. Clinical, demographic, and laboratory data were recorded, and a fasting abdominal ultrasound was carried out to diagnose NAFLD and identify gallstones. SPSS version 26.0 (IBM Corp., Armonk, NY) was used for data analysis, and a p-value of less than 0.05 was considered statistically significant.

Results

Among 246 patients with NAFLD, 152 (62%) were women. The mean age was 48.3 ± 10.2 years. Gallstones were diagnosed in 55 (22.4%) of the patients. Advanced age (p = 0.013), female gender (p = 0.037), increased gamma-glutamyl transpeptidase (GGT) levels (p = 0.029), and increased triglyceride levels (p ≤ 0.01) were significantly associated with gallstones.

Conclusion

This study points toward a high co-occurrence of gallstone disease in NAFLD patients, which is 22.4%. Major risk factors were female gender, old age, hypertriglyceridemia, and increased GGT levels. Timely detection and specific screening for gallstones in patients with NAFLD may reduce biliary complications and also lead to better clinical outcomes. It is advisable to conduct multicenter prospective studies for the generalizability of the findings of this study.

## Introduction

Globally, non-alcoholic fatty liver disease (NAFLD) has emerged as one of the most commonly recognized liver-related disorders in recent times, affecting 25-30% of the adult population [1,2]. NAFLD is characterized by the liver fat accumulation in the absence of substantial alcohol consumption and encompasses a range of conditions from simple steatosis through non-alcoholic steatohepatitis (NASH) that progresses further through fibrosis, cirrhosis, and even hepatocellular carcinoma [3,4].

Increased rates of obesity, physical inactivity, and type 2 diabetes mellitus (T2DM) are major contributors to the growing burden of NAFLD, most notably in developing nations such as Pakistan, where studies in recent years described a range of prevalence from 14% up to as high as 30% [5]. Likewise, gallstone disease (GSD), or cholelithiasis, is one of the most frequent biliary pathologies worldwide, occurring in 10-20% of adults [6]. Cholesterol stones are the main components of gallstones, whose pathogenesis is heavily influenced by metabolic determinants, i.e., dyslipidemia, obesity, insulin resistance, and weight loss [6,7]. Of note, most of these risk factors are also risk factors for NAFLD, implying a pathophysiologic association between the two diseases.

Various studies have shown the association of NAFLD with the presence of gallstones. Liu et al. (2014) identified an increased prevalence of gallstones in Chinese female patients with NAFLD [8]. Similarly, Ahmed et al. observed an increased frequency of gallstones in young patients with NAFLD [9]. Another study done by Ahmed et al. in the Pakistani population showed a significant association of metabolic syndrome with the presence of both gallstones and NAFLD, with a significant association of family history of cholelithiasis with the presence of gallstones in the NAFLD population [10]. The same has been shown by a cohort study in Italy by Fracanzani et al. (2012), reporting a frequency of gallstones in NAFLD cases of 20% [11]. These studies indicate that metabolic alterations frequently encountered in NAFLD, i.e., hypertriglyceridemia and insulin insensitivity, lead to bile supersaturation with cholesterol, resulting in stone formation. This tendency is further augmented by gallbladder hypomotility, a condition frequently encountered in obese subjects as well as in metabolic syndrome patients [12].

NAFLD and gallstones are both common health problems in Pakistan, as a result of increased urbanization, sedentary dietary lifestyles filled with fats and carbohydrates, as well as genetic susceptibility toward metabolic syndrome. However, local data concerning the frequency of gallstones in NAFLD are scarce. Recognizing the coexistence of NAFLD with gallstones is important, as the combination of the two can make the management clinically challenging. Therefore, this study aimed to determine the frequency of gallstones in patients diagnosed with NAFLD and also compared the baseline clinical, biochemical, and metabolic characteristics between those with and without gallstones.

## Materials and methods

After obtaining approval from the Ethical Review Committee, Sindh Institute of Urology and Transplantation (SIUT-ERC-2022/A-671), this cross-sectional observational study was carried out at the Department of Hepatogastroenterology and General Surgery, Sindh Institute of Urology and Transplantation, from January 1, 2023, to June 30, 2023. After taking informed consent, all adult patients aged ≥18 years with the presence of fatty liver on ultrasound were enrolled in the study. While, patients with history of significant alcohol consumption (>20 g/day for women and >30 g/day for men), those with history of liver disease (e.g., viral hepatitis, autoimmune hepatitis, and hemochromatosis), patients with prior history of cholecystectomy, pregnant females, and patients on medications known to influence the lipid metabolism (e.g., statins and fibrates) were excluded from the study.

Assuming a prevalence of gallstones in NAFLD patients to be approximately 20%, with a 5% margin of error and a 95% confidence interval, the minimum required sample size was calculated to be 246 patients.

Data collection

The demographic data were recorded from each patient at the time of enrollment, including clinical history and physical examination findings. Laboratory parameters such as hemoglobin, total leukocyte count (TLC), platelet count, serum bilirubin, aspartate transaminase (AST), alanine transaminase (ALT), gamma-glutamyl transpeptidase (GGT), alkaline phosphatase, low-density lipoprotein (LDL), high-density lipoprotein (HDL), and serum triglyceride levels were also recorded at the time of enrollment. Fasting abdominal ultrasonography was performed by an expert radiologist with more than 10 years of experience in hepatobiliary diseases to confirm the diagnosis of NAFLD (presence of hyper-echoic liver) and to detect the presence of gallstones.

Data analysis procedure

Data were entered and analyzed using SPSS version 26.0 (IBM Corp., Armonk, NY). Continuous variables were expressed as mean ± standard deviation, while categorical variables were expressed as frequencies and percentages. Independent t-tests were used to compare continuous variables between NAFLD patients with and without gallstones, while chi-square tests were applied for categorical variables. A p-value <0.05 was considered statistically significant.

## Results

A total of 246 patients were enrolled in the study. Among them, 152 (61.8%) patients were females. The most common presenting complaint was right hypochondrial pain observed in 79 (32.1%) patients, followed by postprandial fullness and nausea in 54 (22%) and 21 (8.5%) patients, respectively. On ultrasound, gallstones were observed in 55 (22.4%) patients (Table 1).

**Table 1:** Demographic data of the categorical variables included in the study (n = 246).

Study population		n (%)
Gender	Males	94 (38.2)
	Females	152 (61.8)
Symptoms of dyspepsia at baseline	Right hypochondrial pain	79 (32.1)
	Postprandial fullness	54 (22)
	Nausea	21 (8.5)
Gallstones	Present	55 (22.4)
	Absent	191 (77.6)

At the time of presentation, the mean age of the study population was 48.3 ± 10.2 years, the mean hemoglobin level was 12.8 ± 1.6 g/dL, the mean TLC was 7.5 ± 2.1 ×10^9^/L, and the average platelet count was 230 ± 68 ×10^9^/L. The mean total bilirubin was 0.8 ± 0.3 mg/dL, AST was 42 ± 18 U/L, ALT was 58 ± 22 U/L, and GGT was 65 ± 28 U/L. Lipid profile analysis showed mean total cholesterol of 205 ± 38 mg/dL, LDL cholesterol of 132 ± 28 mg/dL, HDL cholesterol of 38 ± 9 mg/dL, and triglycerides of 188 ± 56 mg/dL (Table 2).

**Table 2:** Demographic data of the continuous variables included in the study (n = 246). TLC: total leucocyte count; ALT: alanine transaminase; AST: aspartate transaminase; GGT: gamma-glutamyl transpeptidase; LDL: low-density lipoprotein; HDL: high-density lipoprotein. Reference ranges - Hemoglobin (g/dl): 14-16 for men and 12-14 for women; TLC (x10^9^/L): 4-11; platelets (x10^9^/L): 250-400; total bilirubin (mg/dl): 0.9-1.2; AST (U/L): up to 40; ALT (U/L): up to 33 for men and 25 for women; alkaline phosphatase (U/L): 105-135; GGT (U/L): up to 50; total cholesterol (mg/dl): less than 200; LDL (mg/dl): less than 110; HDL (mg/dl): 40-60; serum triglycerides (mg/dl): less than 250.

Study population	Mean ± SD
Mean age (years)	48.3 ± 10.2
Hemoglobin (g/dl)	12.8 ± 1.6
TLC (x10^9^/L)	7.5 ± 2.1
Platelets (x10^9^/L)	230 ± 68
Total bilirubin (mg/dl)	0.8 ± 0.3
ALT (U/L)	58 ± 22
AST (U/L)	42 ± 18
Alkaline phosphatase (U/L)	102 ± 35
GGT (U/L)	65 ± 28
Total cholesterol (mg/dl)	205 ± 38
Serum LDL (mg/dl)	132 ± 28
Serum HDL (mg/dl)	38 ± 9
Serum triglycerides (mg/dl)	188 ± 56

On comparative analysis, factors associated with the presence of gallstones in the NAFLD population were advanced age (p = 0.013) (Table 3), female gender (p = 0.037), and raised GGT (p = 0.029) and triglyceride levels (p ≤ 0.01). However, no statistically significant association was observed between the gallstones and other lab parameters such as hemoglobin, TLC, platelet count, total bilirubin, ALT, LDL, HDL, or total cholesterol levels (Table 4).

**Table 3:** Comparison of categorical variables in predicting gallstones in non-alcoholic fatty liver disease (n = 246). * The chi-square test was used for the calculation of p-values of categorical variables.

Variable		Gallstones		Chi-square value*	p-value
		Present (N = 55), N (%)	Absent (N = 191), N (%)		
Gender	Male	16 (29)	78 (40.8)	4.22	0.04
	Female	39 (71)	113 (59.2)		

**Table 4:** Comparison of continuous variables in predicting gallstones in patients with non-alcoholic fatty liver disease. * The t-test was used for the calculation of p-values of continuous variables. TLC: total leucocyte count; ALT: alanine transaminase; AST: aspartate transaminase; GGT: gamma-glutamyl transpeptidase; LDL: low-density lipoprotein; HDL: high-density lipoprotein. Reference ranges - Hemoglobin (g/dl): 14-16 for men and 12-14 for women; TLC (x10^9^/L): 4-11; platelets (x10^9^/L): 250-400; total bilirubin (mg/dl): 0.9-1.2; AST (U/L): up to 40; ALT (U/L): up to 33 for men and 25 for women; alkaline phosphatase (U/L): 105-135; GGT (U/L): up to 50; total cholesterol (mg/dl): less than 200; LDL (mg/dl): less than 110; HDL (mg/dl): 40-60; serum triglycerides (mg/dl): less than 250.

Variable	Gallstones		t-test value*	p-value
	Present (N = 55), Mean ± SD	Absent (N = 191), Mean ± SD		
Mean age (years)	51.4 ± 9.8	47.3 ± 10.1	2.58	0.01
Hemoglobin (g/dl)	12.7 ± 1.5	12.8 ± 1.6	-0.56	0.58
TLC (x10^9^/L)	7.6 ± 2.0	7.5 ± 2.1	0.331	0.74
Platelets (x10^9^/L)	227 ± 70	231 ± 67	-0.43	0.66
Total bilirubin (mg/dL)	0.8 ± 0.3	0.8 ± 0.3	0.22	0.82
ALT (U/L)	32 ± 18	28 ± 16	0.11	0.09
AST (U/L)	43 ± 17	41 ± 16	0.43	0.48
Alkaline phosphatase (U/L)	105 ± 38	101 ± 34	0.85	0.89
GGT (U/L)	72 ± 30	61 ± 27	2.33	0.02
Total cholesterol (mg/dL)	208 ± 40	204 ± 37	0.73	0.47
Serum LDL (mg/dL)	134 ± 39	131 ± 27	0.85	0.39
Serum HDL (mg/dL)	37 ± 9	39 ± 11	-0.98	0.32
Serum triglycerides (mg/dL)	201 ± 54	184 ± 46	2.04	0.04

## Discussion

The current study was conducted to assess the prevalence of gallstones in patients diagnosed with NAFLD in a tertiary care facility in Pakistan and then to compare the clinical, biochemical, and metabolic profiles between NAFLD patients with and without gallstones.

On analysis, gallstones were detected in 22.4% of cases of NAFLD, which reflects the significant co-occurrence of these two metabolic conditions in the study population. The observed percentage of the presence of gallstones is in accordance with the internationally reported data, and it also reflects the increasing awareness of the common pathophysiology among GSD and NAFLD. The 22.4% gallstone prevalence in this study is similar to that reported by Fracanzani and his colleagues, in which 20% of their NAFLD population was documented to be presenting with gallstone disease, thus validating that gallstone disease and NAFLD tend to coexist across geographical regions [11]. A study done by Liu and his colleagues also reported a higher occurrence of gallstones among Chinese female patients with NAFLD, highlighting a demographic trend that was also replicated in this study, where female gender was also significantly related to the occurrence of gallstones (p = 0.037) [8]. The female predominance in our group can be explained by hormonal effects like estrogen that favor cholesterol supersaturation of bile and decreased gallbladder motility, which can allow gallstone formation [13].

Our findings also align with the local data of Ahmed et al., in which there was a significant correlation between the components of the metabolic syndrome and the occurrence of gallstones among NAFLD patients in the population of Pakistan [10]. These results also reinforced the importance of significant family history and genetic makeup, causing alterations resulting in increased formation of gallstones, a finding also reinforced by the strong associations in this study between increased triglyceride levels and gallstone presence (p ≤ 0.01). Hypertriglyceridemia, the defining feature of the metabolic syndrome, leads to cholesterol supersaturation of bile, eventually leading to lithogenesis [14].

In our study, we observed that the age of patients with NAFLD bearing gallstones was significantly greater in comparison to their counterparts (p = 0.013). Age is known to be a risk factor for gallstone generation, given that greater age correlates with cumulative exposure to biliary insults, gallbladder hypomotility, and dysfunction of bile composition [15]. Such a trend had consistently appeared in previous studies, including the cohort by Ahmed et al., where younger individuals with NAFLD had a reduced gallstone occurrence in comparison to their elderly counterparts [9].

Biochemically, higher levels of liver enzymes like GGT (p = 0.029) were also strongly related to gallstones in our population. Increased GGT levels, which are also markers of cholestasis and oxidative stress, further indicate a possible overlap between biliary disease and hepatic steatosis [16]. Similarly, elevated triglyceride levels were also found to be significantly correlated with gallstones in this study, yet other parameters of the lipid profile, like LDL, HDL, and total cholesterol, did not differ significantly. This is contrary to a few previous studies in that low HDL cholesterol was previously reported as one of the risk factors for gallstones in NAFLD patients [17]. Our study's lack of strong association may be attributable to the population differences or to confounding factors that may include dietary habits and genetic susceptibility specific to the Pakistani population.

We acknowledge that there are certain limitations that can be attributed to our study. To begin, it is single-centered and cross-sectional in design, which may fall short in representing the complete epidemiological profile of the nation. Secondly, a small sample size may limit the generalizability of our results to the whole population. Third, we did not evaluate other risk factors that might include dietary behaviors, physical inactivity, genetic susceptibility, or family history of gallstone disease that may give a better risk profile. Finally, the lack of a non-NAFLD control group constrains the direct determination of the relative risk of gallstones attributable to NAFLD alone. Multi-centered prospective studies in the future are needed to confirm and extend the present results.

## Conclusions

This study showed a significant prevalence of gallstones among patients with NAFLD in a tertiary care center in Pakistan, proving the significant co-occurrence of these two metabolic diseases. These results are in accordance with both global and local literature, which point toward the common pathophysiologic mechanisms of hypertriglyceridemia, metabolic syndrome, and gallbladder hypoactivity that lead to both gallstone disease and NAFLD. Interestingly, old age, female sex, raised levels of GGT, and increased levels of triglycerides were the factors associated with the presence of gallstones among the population of patients with NAFLD. These observations point toward increased vigilance and specific screening in the population of NAFLD patients, particularly among those presenting with these risk indicators. Recognition of gallstone disease in patients with NAFLD early in the course of the disease may prove to be crucial in optimizing outcomes and lowering the biliary complication burden in this population.
